# MESENTERIC CYST: ABDOMINAL LYMPHANGIOMA

**DOI:** 10.1590/S0102-67202014000200016

**Published:** 2014

**Authors:** Diogo Gontijo Dos REIS, Nícollas Nunes RABELO, Sidnei Jose ARATAKE

**Affiliations:** 1 Serviço de Ortopedia da Faculdade Atenas (Orthopedics Service of Paracatu Atenas School), Paracatu, MG, Brazil.; 2 Serviço de Cirurgia Geral do Hospital São Francisco (Service of General Surgery, San Francisco Hospital), Taquaral, GO, Brazil.

## INTRODUCTION

Mesenteric cyst is defined as a cystic lesion located between the leaflets of the mesentery from the duodenum to the rectum , being most commonly found in ileum level. Since its first description in 1507 by Benevienae until 1993 there are only about 820 cases reported in the literature^4-6^.

Lymphangiomas are benign tumors, probably congenital, are more common in the cervical and axillary regions. They are unusual in abdominal and pancreas location. Its incidence is estimated at around 1:100,000 and 1:20,000 admissions in adults and in children. The first excision was performed by Tillaux (quoted Chung) only in 1802^5^. Despite the long recognition of this disease, its origin classification and pathology remain controversial. The highest incidence is between the third and fourth decades of life, with 75% of those diagnosed after ten years with a slight female predominance. The term lymphangioma is appropriately used when there is hemodynamic isolation, or the injury is not related to the blood system^10-13^. Lymphangiomas are a major group of so-called vascular hamartomas, which result from a failure in the evolutionary development of the vascular system, including lymphatic and/or arteries and veins^3^.

These lymphatic tumors are divided in: 1) simple, with capillary lymphatic channels; 2 ) cavernous, with dilated lymphatics and the presence of capsule; and 3) macrocystic malformations, clinically termed "cystic hygroma"^6^. This is the most common type, and the most affected sites are head and neck. The main differential diagnosis is hemangioma, branchial cysts, lipomas and rabdmiossarcoma. Diagnosis is made by biopsy of suspicious nodules, and the main treatment is surgical excision.

## CASE REPORT

**Figure 1:**
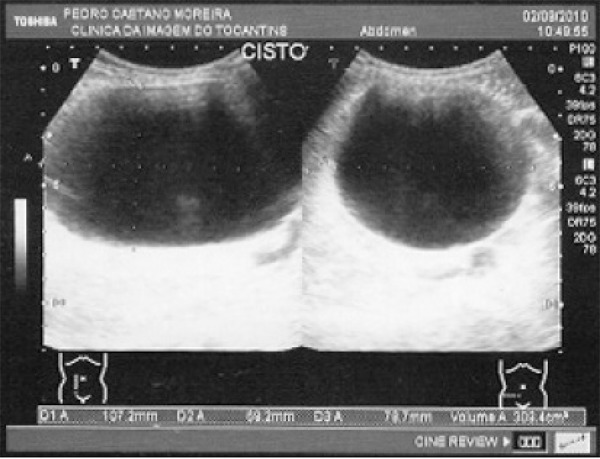
Ultrasound image of the abdomen with intraperitoneal cyst

Fifty-eight years man came to San Francisco Hospital in Taquaral of Goiás, Brazil, after previous medical care without diagnosis or treatment. He had a history of a large mass over a year of slow evolutionary character. He denied pain, headache, fever, diarrhea and other symptoms. Physical examination revealed good general condition, acyanotic, afebrile, hydrated, normotensive, rhythmic, symmetrical heart sounds without murmur. His abdomen had a moving mass of 10 cm in diameter, painless to superficial and deep palpation. Ultrasonography showed an intraperitoneal cyst apparently mesenteric (Figure 1).

**Figure 2:**
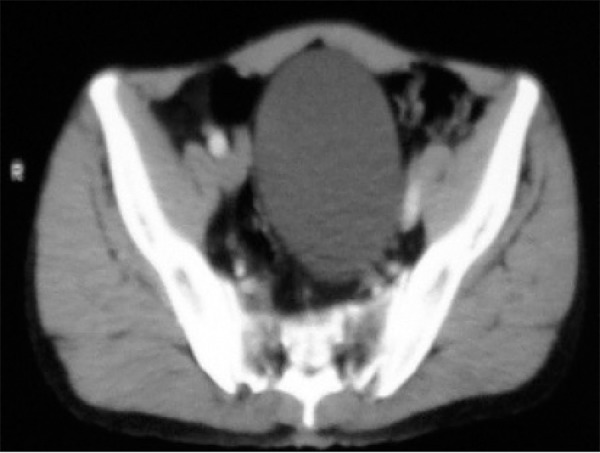
CT scan with contrast of the abdomen showing the median cystic image

**Figure 3:**
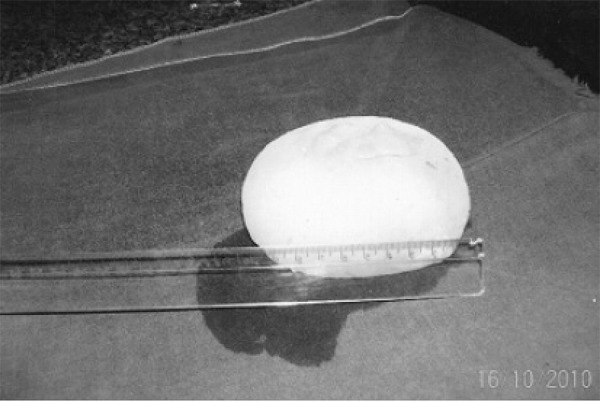
Resected cyst (lymphangioma)

With the uncertain diagnosis was conducted to computed tomography of the abdomen reviling a hyperdense mass of cystic aspect, not contrasted, measuring 9.0x7.1 cm in its greatest transverse axis located in the hypogastrium, without being adherent to the bladder (Figure 2). Was opted to indicate exploratory laparotomy through supra-umbilical median approach. Was encountered an intraperitoneal cyst in the region of retouterine recess (Douglas), totally free and avascular (Figure 3). Histopathologic examination was a mesenteric cyst/lymphangioma. The patient was discharged without intercurrences.

## DISCUSSION

There are no pathognomonic signs and symptoms of mesenteric cyst; However, paper presented by Santana et al.^11^ reporting 18 cases showed abdominal pain and mass (72%), vomiting and constipation; one patient presented with acute abdomen pain. Palpation usually presents itself painless, smooth contour and well defined with mobility in the transverse direction and around its axis (Tillaux signal)^2-11^ The increase in abdominal volume is slow, progressive and late noticed in some cases, mingling with ascites in about 18-20%. There are few reports of malignant mesenteric cysts, usually low-grade sarcomas. Kurtz et al. reviewed 162 cases and found only 3% of malignant transformation, all in adults. Are incidental findings during laparotomy or imaging, up to 40 % of cases. Acute abdomen occurs when there is rupture, infection, bleeding or twisting of the cyst, and confused with appendicitis or aortic aneurysm.

Laboratory tests little help the diagnosis. Simple X-rays of the abdomen may show calcifications; arteriography and intestinal transit may show compressive mass. However, ultrasonography, computed tomography computed and magnetic resonance imaging are the exams that provide better diagnosis.

Once diagnosed, all mesenteric cyst should be resected in order to avoid their complications^2-11^, recurrence, malignant transformation and possible complications (hemorrhage, torsion, obstruction, traumatic rupture and infection)^8-12^. Internal drainage may be an option when there is possibility of short bowel syndrome. In selected cases laparoscopic approach can be used^13-15^.

Santana et al.^11^ classified them as pathologically serous, bloodserous, chylous, with blood. In this case hydatid cist was also placed on judgment in the differential diagnosis, before the end of lymphangioma.
